# Low translational and rotational movements with 2-point stainless-steel retainers over a period of 1 and 3 years

**DOI:** 10.1007/s00056-023-00505-y

**Published:** 2023-12-28

**Authors:** Sarah Koller, Christian Niederau, Irma Azraq, Rogerio Bastos Craveiro, Isabel Knaup, Michael Wolf

**Affiliations:** 1 Department of Orthodontics, Dental Clinic, University Hospital RWTH Aachen, Pauwelsstr. 30, 52074 Aachen, Germany; 2 Orthodontic Specialist Practice Dr. Inge Kiegel-Koller, Bergheim, Germany

**Keywords:** Fixed orthodontic appliances, Retention stability, Changes in dental occlusion, Retention protocol, Treatment outcome, Festsitzende kieferorthopädische Apparaturen, Retentionsstabilität, Veränderungen der dentalen Okklusion, Retentionsprotokoll, Therapieergebnis

## Abstract

**Objectives:**

Long-term stabilization of orthodontic treatment outcomes is an everyday challenge in orthodontics. The use of permanently attached lingual retainers has become gold standard. However, in some cases, patients with fixed lingual retainers show retainer-associated side effects. Aiming to reduce these side effects, clinical knowledge about how tooth and arch form stability adaption takes place over time is important to improve long-term retention protocols. Therefore, the present study aimed to investigate occlusion stability and risks for a newly developing malocclusion in a time-dependent manner in patients being treated with permanent 2‑point steel retainers.

**Materials and methods:**

In this retrospective cohort study, a total of 66 consecutive patients with round stainless-steel retainers were analyzed for postorthodontic occlusion changes after 1 year (group 1, *n* = 33) and 3 years (group 2, *n* = 33). Digital Standard Tessellation Language (STL) datasets of the lower jaw were obtained before retainer insertion (T0), and after a 1- (T1) or 3‑year (T2) retention period. Using superimposition software, T1 and T2 situations were compared to T0 regarding rotational and translational changes in tooth positions in all three dimensions.

**Results:**

Occlusion changes were low in both groups. The investigated lower canines were nearly stable in the 1‑ and 3‑year group, although a retention-time-dependent increase in tooth position change of the central and lateral incisors could be observed.

**Conclusion:**

The present data provide evidence for time-dependent development of posttherapeutic occlusal adaption limited to central and lateral incisors in patients treated with a 2-point retainer. The observed occlusal changes should be interpreted as an occlusal adaption process rather than severe posttreatment changes associated with the orthodontic retainer.

## Abstract (de)

**Zielsetzung:**

Die langfristige Stabilisierung von kieferorthopädischen Behandlungsergebnissen ist eine alltägliche Herausforderung in der Kieferorthopädie. Die Verwendung von festsitzenden lingualen Retainern hat sich als Goldstandard etabliert. In einigen Fällen treten jedoch bei Patienten mit festsitzenden Lingualretainern retainerassoziierte Nebenwirkungen auf. Das klinische Wissen darüber, wie sich die Stabilität der Zähne und des Zahnbogens im Laufe der Zeit anpasst, ist von erheblicher Relevanz, um diese Nebenwirkungen zu verringern und die Langzeit-Retentionsprotokolle zu verbessern. Ziel der vorliegenden Studie war es daher, die Okklusionsstabilität und die Risiken für eine sich neu entwickelnde Malokklusion in Abhängigkeit von der Zeit bei Patienten zu untersuchen, die mit permanenten 2‑Punkt-Stahlretainern behandelt werden.

**Material und Methode:**

In dieser retrospektiven Kohortenstudie wurden insgesamt 66 aufeinanderfolgende Patienten mit runden Edelstahl-Retainern auf postkieferorthopädische Okklusionsveränderungen nach einem Jahr (Gruppe 1, *n* = 33) und 3 Jahren (Gruppe 2, *n* = 33) untersucht. Digitale STL (Standard Tessellation Language)-Datensätze des Unterkiefers wurden vor dem Einsetzen des Retainers (T0), nach einem (T1) oder nach 3 Jahren (T2) Retentionszeit erstellt. Mithilfe einer Überlagerungssoftware wurden die T1- und T2-Situationen hinsichtlich der Rotations- und Translationsänderungen der Zahnpositionen in allen 3 Dimensionen mit T0 verglichen.

**Ergebnisse:**

Veränderungen in der Okklusion waren in beiden Gruppen gering. Die untersuchten unteren Eckzähne waren in der Ein-Jahr- und in der Drei-Jahre-Gruppe nahezu stabil, obwohl bei den zentralen und seitlichen Schneidezähnen eine von der Retentionszeit abhängige Zunahme der Zahnpositionsveränderung beobachtet werden konnte.

**Schlussfolgerung:**

Die vorliegenden Daten liefern Beweise für eine zeitabhängige Entwicklung der posttherapeutischen okklusalen Adaptation, die sich auf die zentralen und lateralen Schneidezähne von Patienten beschränkt, die mit einem 2‑Punkt-Retainer behandelt wurden. Die beobachteten okklusalen Veränderungen sollten eher als okklusaler Anpassungsprozess denn als schwerwiegendes posttherapeutisches Rezidiv im Zusammenhang mit dem kieferorthopädischen Retainer interpretiert werden.

## Introduction

One of the greatest challenges in modern orthodontics is the stabilization of treatment outcomes. Various studies have reported a relapse particularly of the lower incisors into their previous malocclusion [17, 29]. Although the reason for this relapse is not yet entirely understood, it appears to be a multifactorial problem caused, for example, by insufficient treatment planning, overexpansion or enormous changes of arch forms, an unfavorable skeletal growth pattern, a lack of stable occlusion, muscular dysfunctions and habits, or resilient Sharpey’s fibers [8, 18, 19, 22]. Patients’ expectations regarding preservation of a lifelong esthetic smile have increased in recent years. Securing the therapy result after years of orthodontic treatment and preventing a relapse of the anterior teeth toward the baseline malocclusion is of highest importance for patients [7]. To retain upper and lower incisors, both permanently fixed retainers and removable appliances are commonly used, e.g., Hawley-type retainers or vacuum-formed splints. In addition to fixed retainers, passive plates have an added impact on the prevention of tertiary crowding [18, 34].

Higher demands for long-term retention devices in recent years have led to increasing requirements on lingual fixed retainers in terms of material stability and economic aspects [30].

Considering suppositional patient’s adherence for long-term stability of treatment outcome, fixed lingual retainers appear to be superior to removable passive plates [7]. According to the literature and to clinical practice, there is evidence that patients with fixed retainers show less overall posttreatment changes after having completed multibracket appliance treatment [1, 5]. However, it is also known that the removal of a fixed retainer can lead to changes in tooth position even after a longer insertion period [10].

Over the last few decades, various retainer systems have been established in orthodontics. Since their introduction by Zachrisson in 1977, multistranded wire retainers are very commonly used and are considered the gold standard in posttreatment retention [6, 11, 35, 36]. Including a bonding site on each incisor, the multistranded wire retainer is beneficial for lower anterior tooth stabilization and preserves the intrinsic mobility of the teeth due to the elastic property of the wire [6, 15, 24, 34]. Multistranded wires can easily be adapted to the anterior tooth segment, but they need to be adjusted precisely to avoid gaps between the wire and the tooth surface. Larger gaps are bridged with composite filling material, which in turn might limit the patient’s comfort and possibly lead to premature contacts and increased plaque accumulation [16, 17].

Various studies reported a higher frequency of bonding side defects, when comparing twistflex to round stainless-steel retainers [24, 26]. In addition, possible residual stress in the wire and iatrogenic forces may lead to unwanted tooth movement, resulting in, for example, loss of torque control (x-effect or twist effect) [4, 6, 28]. Apart from these side effects, defects of bonding sites are often noticed by the patients only after relapse of anterior tooth positions into malocclusion [23].

These impacts led to the introduction of innovative computer-aided designed and computer-aided manufactured (CAD/CAM) retainers, such as Memotain (CA Digital GmbH, Hilden, Germany) or prime4me® RETAIN3R (Dentaurum, Ispringen, Germany). These high precision retainers are bonded to the lingual surfaces of the upper or lower incisors [13, 14, 33]. However, a high level of production complexity affects the cost-effectiveness of these high-precision CAD/CAM retainers.

In order to primarily maintain the intercanine distance, 2‑point stainless steel retainers are considered a cost-efficient alternative to CAD/CAM retainers [6]. In contrast to modern CAD/CAM and multistranded wire retainers, 2‑point stainless steel retainers are bonded to the canines only, which has three main advantages: first, by enabling interproximal cleaning, an improvement of oral hygiene can be observed [3, 34]. Oral hygiene is simplified by the fact that dental floss only needs to be passed under the retainer once and can then, due to the absence of further adhesive points, be easily moved to the next tooth. Second, the failure rate of bonding site defects was described to be significantly lower compared to the multistranded wire retainers, with the latter being bonded to all anterior teeth [3, 27, 31]. Possible defects of 2‑point retainers can be immediately noticed by the patients [23]. Third, though rotational movements of lower incisor were noticed [23, 31], no restrictions of intrinsic tooth mobility were observed due to the passive retention character [3]. On the other hand, the high wire diameter of the retainer could induce patient discomfort and tends to be unsuitable for the upper jaw [27, 31]. In addition, it remains unclear to what extent a retainer, which is only attached to the canines, provides sufficient retention for the incisors over time in order to provide an optimal posttreatment retention protocol.

Up to now, there are no studies analyzing the stability of 2‑point retainers on translational and rotational movements and there is no information whether posttreatment changes in tooth position occur early within the first years of retention or later. Thus, the aim of the present study was to systematically analyze tooth position stability of patients being treated with 2‑point retainers using a digital surface-matching approach. To address the question whether potential posttreatment tooth movements occur directly after retainer placement or later, we analyzed the translational and rotational movements of the lower anterior teeth during a retention period of 1 and 3 years [21].

## Methods

### Patients

A total of 66 fixed round stainless-steel retainers, which had been inserted to stabilize preceding active orthodontic treatment outcome, were analyzed 1 year (group 1; *n* = 33) and 3 years (group 2; *n* = 33), respectively, after intraoral insertion. The first group included 20 female and 13 male patients with retainers 1.4 years on average in situ. The second group included 22 females and 11 males with retainers 3.4 years on average in situ. The inclusion criteria were completed fixed orthodontic treatment with a secured class I occlusion, 2‑point stainless steel retainer in combination with a Hawley retainer.

Consecutive patients following preceding treatment with braces including at least one attendance at the orthodontic practice each year were included in this study. Patients were randomly distributed into the two groups. According to our clinical protocol, cases with < 7 mm crowding and treated without extractions were included. None of the patients had bonding-site defects or retainer failure. The study was approved by the Ethics Committee of the University of Aachen (EK 232-20). The study was conducted with informed consent of all patients and in full accordance with the Declaration of Helsinki (2008).

### Retainer insertion

**Fig. 1:**
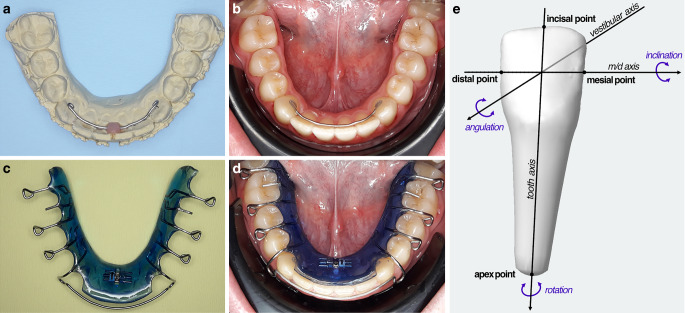
Photographs of **a** 2-point retainer bent by a technician attached to a plaster model and **b** in situ with adhesive attachment to the canines; **c** Hawley retainer and **d** Hawley retainer in situ. **e** Schematic of the coordinate system with reference points and axes Fotos. **a** Von einem Techniker gebogener 2‑Punkt-Retainer, befestigt auf einem Gipsmodell, **b** 2-Punkt-Retainer in situ, mit Klebebefestigung an den Eckzähnen, **c** Hawley-Retainer und **d** Hawley-Retainer in situ. **e** Schema des Koordinatensystems mit Referenzpunkten und Achsen

All participants in the clinical trial were assessed for eligibility by the same clinician. Following active orthodontic treatment, a round stainless-steel retainer (remanium 0.7 mm/28, round, hard, stainless steel, Dentaurum, Ispringen, Germany) was bonded to both lower canines. Additional passive Hawley retainers were inserted according to the local retention protocol. The labial arch of the Hawley retainers was designed with passive contact to the lower incisors and canines. This contact was not supported by an individual plastic coating (Fig. 1). The patients were instructed to wear the Hawley retainers at night. All retainers were fabricated on plaster models by the same technician in the laboratory of the orthodontic practice after taking impressions of the upper and lower jaw. They were initially stabilized using three strands of dental floss and bonded with Transbond XT (3M Unitek, Breckerfeld, Germany) and GC Ortho Connect Flow (GC Orthodontics, Leuven, Belgium) after conditioning the enamel surfaces for 60 s with 37% phosphoric acid.

### Digitalization and superposition of the dental models

**Fig. 2:**
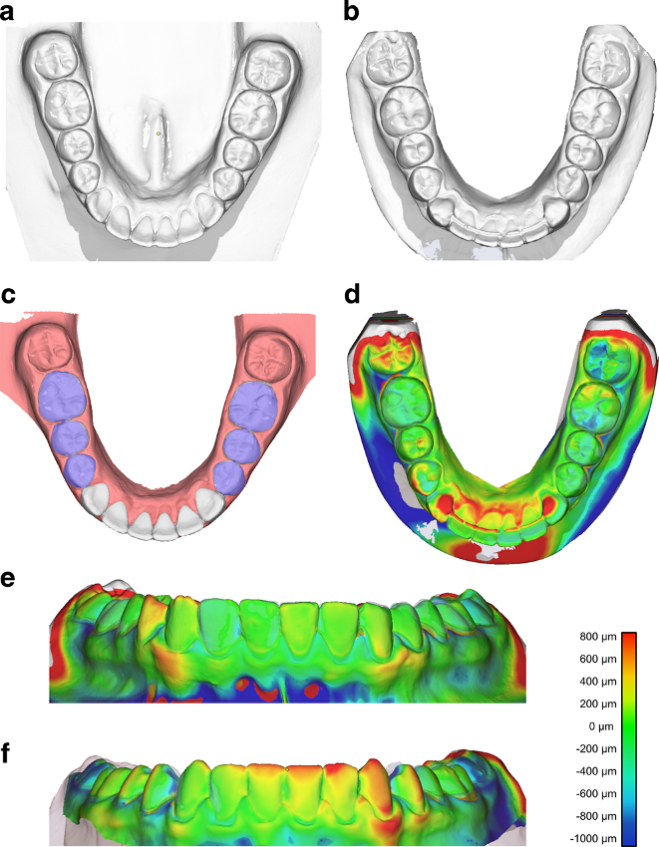
Schematic illustration of the superimposition and three-dimensional (3D) analysis. **a** Digital jaw models before retainer insertion and **b** after 1 or 3 years of retention were superimposed. **c** Both premolars and the first molar were used as reference structures. **d** The 3D overlay dataset was analyzed regarding rotational and translational tooth movements of the anterior tooth segment. **e**,**f **Representative data after 1 or 3 years of retention Schematische Darstellung der Überlagerung und dreidimensionale (3-D) Analyse. **a** Digitale Kiefermodelle vor dem Einsetzen des Retainers und **b** nach einem bzw. 3 Jahren Retention wurden überlagert. **c** Die beiden Prämolaren und der erste Molar wurden als Referenzstrukturen verwendet. **d** Der 3‑D-Overlay-Datensatz wurde hinsichtlich der rotatorischen und translatorischen Zahnbewegungen des Frontzahnsegments ausgewertet. **e**,**f **Repräsentative Daten nach 1 bzw. 3 Jahren Retention

Following debonding of the active orthodontic appliance, impressions were taken of all patients included in the study. These impressions were used to manufacture dental models of each patient (T0 models) before insertion of the round stainless-steel retainers (Fig. 2). Consecutive impressions were repeated after 1 year (T1—group 1) and 3 years (T2—group 2). The T0, T1, and T2 plaster models were manufactured and digitized with a dental model scanner (orthoX® scan, Dentaurum, Ispringen, Germany). Using Standard Tessellation Language (STL) datasets, the digital impressions were transferred to three-dimensional (3D) processing software (OnyxCeph, Image Instruments, Chemnitz, Germany).

Further analysis of the models using a 3D processing software included separation of the teeth at the T0 models. In contrast to the upper jaw, for the mandible no stable anatomical structures can be identified [21, 32]. For this reason, subsequent superposition of T0/T1 and T0/T2 models was accomplished by application of the best-fit method, using premolars and the first molars as reference structures. Then, the movements of the anterior teeth between T0/T1 (group 1) and T0/T2 (group 2) were calculated (Fig. 2).

### Analysis of tooth movements

Changes in comparison with the T0 model regarding rotational and translational tooth movements in the lower anterior region from T1 (1 year) and T2 (3 years) were determined in all three dimensions using the software OnyxCeph (Image Instruments, Chemnitz, Germany). For the analysis of tooth movements, the software determined an individual non-Cartesian (oblique-angled) coordinate system for each tooth with a center located in the tooth crown (Fig. 1e). The rotational variations of tooth position (measured in arc degree, °) were analyzed regarding inclination, rotation, and angulation.

Changes of inclination were determined as a rotation around the “m/d axis” (defined by the mesial and distal contact points). Increased inclination means a movement of the incisal reference point in the buccal direction. Rotational movements were calculated as a rotation around the “tooth axis” (defined by the apex point and the middle between the mesial and distal contact points). Positive values for a rotation indicate movement of a labial reference point to the mesial. Changes of angulation were determined around the vestibular axis which was defined perpendicular to the “m/d axis” and the “tooth axis”. An increase of the values for angulation indicate a movement of the incisal reference point to mesial.

The translational deviations (measured in millimeter) were detected in mesio-distal (along the “m/d axis”, positive values indicate a mesial movement), oro-vestibular (along the “vestibular axis”, positive values for movements to the vestibular) and vertical direction (along the “root axis”, positive values for movements in the apical direction).

To determine a degree of clinical severity, rotational measurements were used to classify treatment outcomes [21]. In brief, based on the clinical appearance of the lower dental arch, at T1/T2, the patients were grouped into three severity groups. Grade 1 indicates mild or no visible changes, grade 2 describes moderate changes, which did not require treatment but were documented to be monitored, and grade 3 refers to severe changes which require treatment. Based on these classification, the changes in tooth position were considered stable if < 5° or < 0.5 mm, moderate if ≥ 5° or ≥ 0.5 mm to ≤ 9° or ≤ 1 mm, and severe if > 9° or > 1 mm irrespective of their directions [32].

### Reproducibility of the data

All superimpositions were performed by one trained and calibrated examiner. To ensure reproducibility of the data, the plaster models of 1 patient were digitalized 10 times with the same protocol and each resulting 3D dataset was analyzed individually. The average measuring difference was < 0.5° and < 0.05 mm.

### Statistical analysis

Significance was determined using Kruskal–Wallis test followed by Dunn’s multiple comparison test or by Mann–Whitney U test in case of a nonparametric distribution identified by Shapiro–Wilk test (GraphPad PRISM, version 9.0.0, GraphPad Software, Insight Partners, New York, NY, USA). Differences in tooth positions were regarded as statistically significant at *p* < 0.05. Absolute values were used for deviation calculation.

## Results

To analyze the potential risk over time for posttherapeutic tooth adaption within the lower anterior teeth treated with a 2-point lingual retainer, analog impressions from 66 patients were digitalized, digitally superimposed and analyzed after 1 year (group 1) and 3 years (group 2) of fixed retention (Fig. 2). Patients of both groups (T1 and T2) still wore the Hawley retainers at the time of evaluation.

### Increased rotational posttherapeutic tooth movement in lower central incisors

**Fig. 3:**
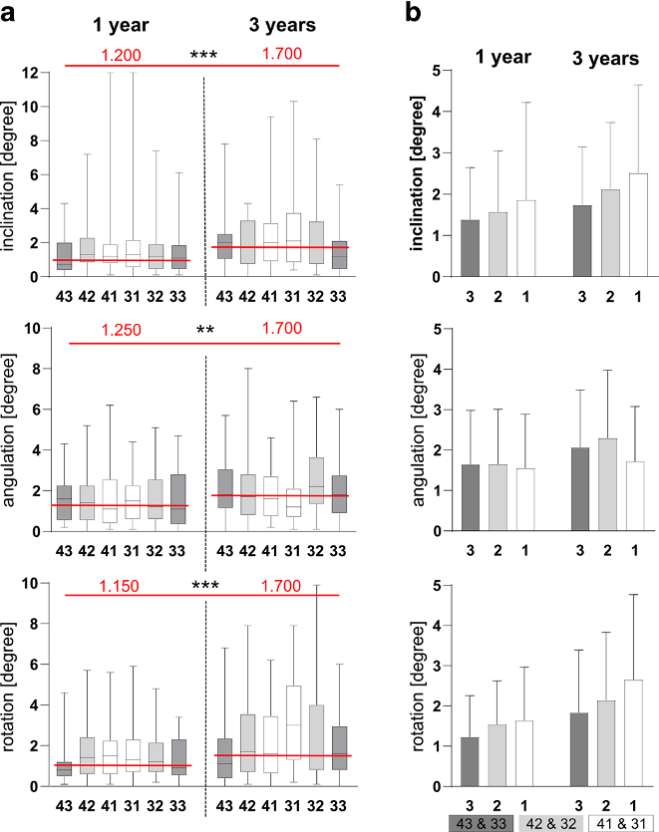
Rotatory changes in tooth position. **a** Deviation of the inclination, angulation, and rotation (in degrees) after 1 and 3 years of retention. The dataset represents 66 (*n* = 66) consecutively analyzed patients 1 year (*n* = 33) and 3 years (*n* = 33) after retainer insertion. **b** Comparison of the individual tooth groups in rotational deviations in degrees. Classification: central incisors (*1*), lateral incisors (*2*), and canines (*3*). *Boxes* show 25th to 75th percentiles, *whiskers* show minimum to maximum values; **p ≤ 0.01, ***p ≤ 0.001 Rotatorische Veränderungen der Zahnstellung. **a** Abweichung der Inklination, Angulation und Rotation in Grad nach einem und 3 Jahren Retention. Der Datensatz repräsentiert 66 (*n* = 66) aufeinanderfolgend untersuchte Patienten ein Jahr (*n* = 33) und 3 Jahre (*n* = 33) nach Einsetzen des Retainers. **b** Vergleich der einzelnen Zahngruppen bei den Rotationsabweichungen in Grad. Gruppierung: zentrale Schneidezähne (*1*), seitliche Schneidezähne (*2*) und Eckzähne (*3*). *Boxen* zeigen 25. bis 75. Perzentile, *Whisker* zeigen Minimal- bis Maximalwerte; **p ≤ 0,01, ***p ≤ 0,001

Visual analyses of the superimposed models demonstrated changes in tooth position in both the 1‑ and 3‑year group. Statistically significant changes were found in all three dimensions for the rotational movements (inclination, angulation, rotation) after 1 and 3 years of retention. The analyzed canines demonstrated less posttherapeutic changes compared to central incisors in inclination and rotation (Fig. 3).

### Increased translational tooth movements after longer retention

**Fig. 4:**
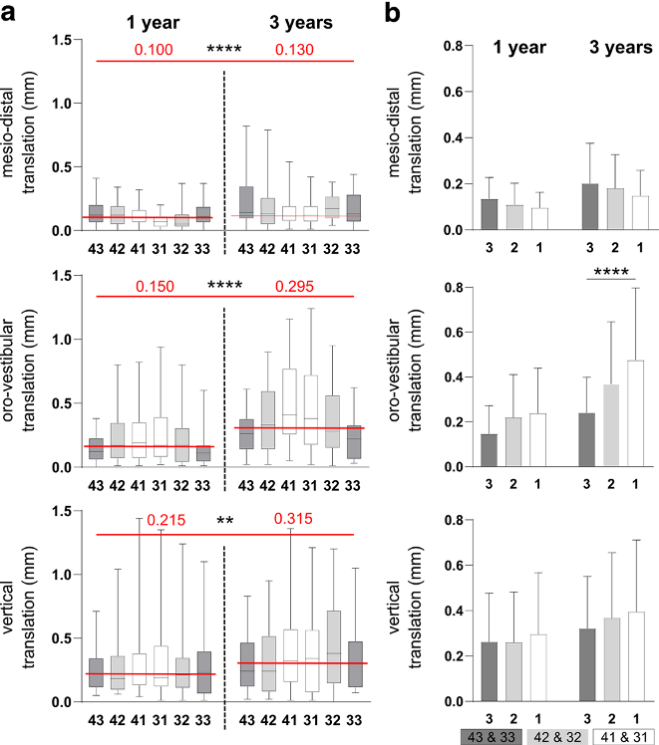
Translational changes in tooth position. **a** Deviation of translational tooth movements (in mm) after 1 and 3 years of permanent retention. The dataset represents 66 (*n* = 66) consecutively analyzed patients after 1 year (*n* = 33) and 3 years (*n* = 33) after retainer insertion. **b** Comparison of the individual tooth groups in translation deviations in mm. Classification: central incisors (*1*), lateral incisors (*2*), and canine (*3*). *Boxes* show 25th to 75th percentiles, *whiskers* show minimum to maximum values; **p ≤ 0.01, ****p ≤ 0.0001 Translatorische Veränderungen der Zahnstellung. **a** Abweichung der translatorischen Zahnbewegungen in mm nach einem und 3 Jahren der permanenten Retention. Der Datensatz repräsentiert 66 (*n* = 66) aufeinanderfolgend analysierte Patienten ein Jahr (*n* = 33) und 3 Jahren (*n* = 33) nach Einsetzen des Retainers. **b** Vergleich der einzelnen Zahngruppen in Translationsabweichungen in mm. Klassifizierung: zentrale Schneidezähne (*1*), seitliche Schneidezähne (*2*) und Eckzähne (*3*). *Boxen* zeigen 25.–75. Perzentile, *Whisker* Minimal- bis Maximalwerte; **p ≤ 0,01, ****p ≤ 0,0001

Regarding transversal translational movements, the greatest changes in tooth position during the retention period were observed for the canines, followed by the lateral incisors and the centrals, for both groups. In the sagittal and vertical direction, the greatest deviation was measured for the central incisors and the lowest changes for the canines. In the longer retention group, changes in tooth position in the sagittal direction were significant for the canines and central incisors (Fig. 4).

### High stability for rotational types of movement in both groups

**Fig. 5:**
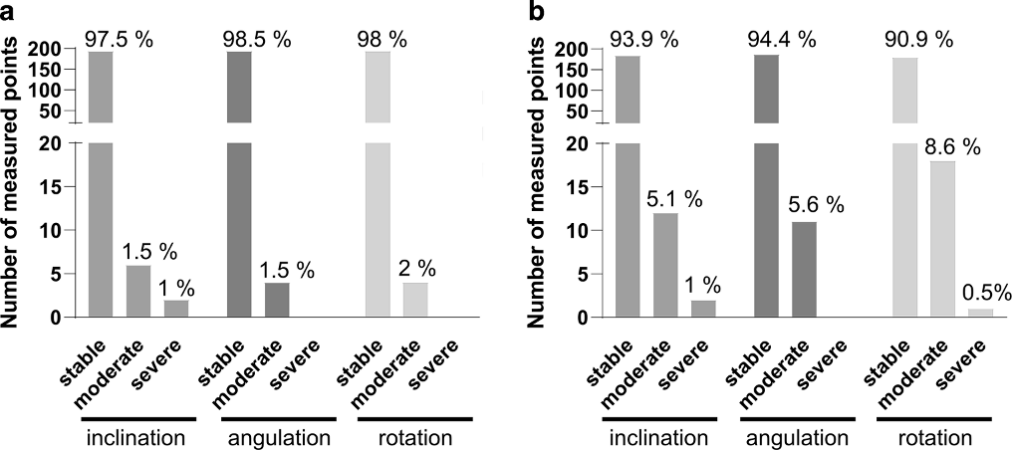
Classification of rotational movements in degrees of clinical severity. The dataset represents 66 (*n* = 66) consecutive analyzed patients **a** 1 year (*n* = 33) and **b** 3 years (*n* = 33) after retainer insertion. According to the extent of tooth inclination, tooth angulation and tooth rotation, the cohort was divided into three different degrees of clinical severity (movement classification: stable: ≤ 5°, moderate: > 5° ≤ 9° and severe: > 9°) Klassifizierung der Rotationsbewegungen nach klinischem Schweregrad. Der Datensatz repräsentiert 66 (*n* = 66) aufeinanderfolgend analysierte Patienten **a** ein Jahr (*n* = 33) und **b** 3 Jahre (*n* = 33) nach Einsetzen des Retainers. Entsprechend dem Ausmaß der Zahnneigung, -angulation und -rotation wurde die Kohorte in 3 verschiedene klinische Schweregrade eingeteilt (Bewegungsklassifikation: stabil: ≤ 5°, moderat: > 5° ≤ 9° und schwer: > 9°)

Overall, a stability of more than 90% was observed in all three rotational motion types (inclination, angulation, rotation) in both the 1‑year and 3‑year groups. Severe changes were very rare at both time points (Fig. 5).

### High stability in transversal translational movements

**Fig. 6:**
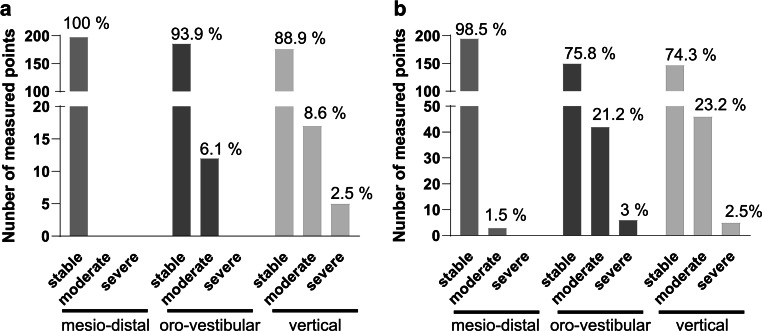
Classification of translational movements in clinical severity. The dataset represents 66 (*n* = 66) consecutively analyzed patients **a** 1 year (*n* = 33) and **b** 3 years (*n* = 33) after retainer insertion. According to the extent of movement, the cohort was divided in three different degrees of clinical severity (movement classification: stable: ≤ 0.5 mm, moderate: > 0.5 mm ≤ 1 mm and severe: > 1 mm) Klassifizierung der Translationsbewegungen nach klinischem Schweregrad. Der Datensatz repräsentiert 66 (*n* = 66) aufeinanderfolgend analysierte Patienten **a** ein Jahr (*n* = 33) und **b** 3 Jahre (*n* = 33) nach Einsetzen des Retainers. Je nach Ausmaß der Bewegung wurde die Kohorte in 3 verschiedene klinische Schweregrade unterteilt (Bewegungsklassifikation: stabil: ≤ 0,5 mm, moderat: > 0,5 mm ≤ 1 mm und schwer: > 1 mm)

After one year of retention, a very high stability of over 85% of the cases was observed for all three translation movements (transversal, sagittal, vertical). The only severe deviations were seen in the vertical direction, where they occurred in 2.5%. After 3 years, a slight overall loss of stability was recorded. The transversal direction remained almost unchanged, whereas stable retention was present in only about 75% of the teeth in the sagittal and vertical directions (Fig. 6). In total, it was found that the canines were the most stable group of teeth regarding sagittal and vertical translations, and the greatest changes were seen in the central and lateral incisors (Table 1).

**Table 1:** Classification of the movements in degrees of severity [26] after 1 and 3 years of retainer treatment, comprising a total of 198 measured points of all three dimensions in rotational movements (tooth inclination, tooth angulation and tooth rotation) and translational movements (horizontal, sagittal, vertical). Tooth movements were separated into individual tooth groups and listed in percentages within each cohort Klassifizierung der Bewegungen in Schweregrade [26] nach einem Jahr und 3 Jahren Retainerbehandlung. Die Tabelle enthält insgesamt 198 Messpunkte in allen 3 Dimensionen für Rotationsbewegungen (Zahnneigung, -angulation und -rotation) und Translationsbewegungen (horizontal, sagittal, vertikal). Die Zahnbewegungen wurden in einzelne Zahngruppen aufgeteilt und in Prozentangaben innerhalb jeder Kohorte aufgeführt

Rotation	Degrees of severity	Inclination			Angulation			Rotation		
		1st incisor	2nd incisor	Canine	1st incisor	2nd incisor	Canine	1st incisor	2nd incisor	Canine
	*1 year*									
	Stable (< 5°)	64 (97%)	64 (97%)	65 (98.5%)	65 (98.5%)	64 (97%)	66 (100%)	63 (95%)	65 (98.5%)	66 (100%)
	Moderate (5–9°)	–	2 (3%)	1 (1.5%)	1 (1.5%)	2 (3%)	–	3 (5%)	1 (1.5%)	–
	Severe (> 9°)	2 (3%)	–	–	–	–	–	–	–	–
	*3 years*									
	Stable (< 5°)	57 (86%)	65 (98.5%)	64 (97%)	64 (97%)	61 (92%)	62 (94%)	56 (85%)	62 (94%)	62 (94%)
	Moderate (5–9°)	7 (11%)	1 (1.5%)	2 (3%)	2 (3%)	5 (8%)	4 (6%)	10 (15%)	3 (4.5%)	4 (6%)
	Severe (> 9°)	2 (3%)	–	–	–	–	–	–	1 (1.5%)	–
Translation	Degrees of severity	Horizontal			Sagittal			Vertical		
		1st incisor	2nd incisor	Canine	1st incisor	2nd incisor	Canine	1st incisor	2nd incisor	Canine
	*1 year*									
	Stable (< 0.5 mm)	66 (100%)	66 (100%)	66 (100%)	59 (89%)	63 (95%)	64 (97%)	58 (87.8)	59 (89%)	59 (89%)
	Moderate (0.5–1 mm)	–	–	–	7 (11%)	3 (5%)	2 (3%)	6 (9.7%)	5 (8%)	6 (9.7%)
	Severe (> 1 mm)	–	–	–	–	–	–	2 (3%)	2 (3%)	1 (1.5%)
	*3 years*									
	Stable (< 0.5 mm)	65 (98.5%)	66 (100%)	64 (97%)	40 (60%)	48 (72%)	62 (94%)	48 (72%)	46 (69.7%)	53 (80.3%)
	Moderate (0.5–1 mm)	1 (1.5%)	–	2 (3%)	20 (30.3%)	18 (28%)	4 (6%)	15 (23%)	19 (28.8%)	12 (18.2%)
	Severe (> 1 mm)	–	–	–	6 (9.7%)	–	–	3 (5%)	1 (1.5%)	1 (1.5%)

## Discussion

Twistflex retainers are the gold standard in fixed retention [36]. Nevertheless, developing technologies allow manufacturing of custom-fabricated retainers using digital CAD/CAM systems. Due to their more precise fit [13], few side effects [14], and their potential impact on oral health [11], they have moved into the focus of modern orthodontics. These reported advantages often go along with high manufacturing effort as well as economic aspects [14]. Therefore, systematically analyzed long-term data have to provide the basis to evaluate modern retention concepts compared to conventional versions. Within this context, the present investigation aimed to provide systematic data on the long-term stability of the widely used 2‑point retainers.

In this study, a digital and user-independent analysis tool which requires expectable stable structures for registration and superimposition was used. In contrast to the palatal rugae in the upper jaw, which are known to be rather stable, such stable nondental structures are not available in the lower jaw [21, 32]. For this reason, molars and premolars had to be used as a reference for superimposing the models. These may undergo vertical settling after debonding, which can influence the precision of the superimposition. To counteract this interference factor, three teeth per side were used in combination with a best-fit registration method. In this manner, individual teeth with increased vertical settling should have influenced the superimposition as little as possible.

Tooth movements in all three spatial planes were investigated regarding translation and rotation under retention in 2‑point retainer cases after short- and long-term retention. Based on recent data indicating that a total of 50% retention loss can be expected 2 years posttreatment [1], it remained unclear which are the most affected teeth, at what time point the development of misalignment could be observed, and whether the misalignment will increase over retention time. The present investigation aimed to address these points by analyzing posttreatment tooth movements after 1 and 3 years of fixed retention. According to the present data, we were able to show only slight changes in tooth position after 1 and 3 years in both translational and rotational tooth movements. For the rotational movements, the observed changes were relatively balanced for each axis. In contrast, the analyzed translational tooth movements showed the largest changes in the vertical direction. This could be explained by the fact that the 2‑point steel retainer does not directly secure the vertical position of the teeth. Overall, canines showed the least rotational and translational movements. This can be explained by the adhesive bonding to these teeth which leads to expectable higher stabilization compared to the nonbonded incisors.

According to data from other published studies on fixed retention systems, the observed posttreatment changes of tooth position for the 2‑point steel retainers were less than for other retainer modifications [23, 31]. A direct comparison of the present data with other retainer studies is limited because in most studies the Little’s irregularity index and not a precise digital measurement system was used [2, 16, 20]. The Little’s irregularity index is more inaccurate and does not allow a statement about all three spatial planes of space and it does not differentiate between rotational and translational tooth movements. This might explain why the studies that applied the Little’s irregularity index method showed larger changes during the retention phase compared to our data [23, 31]. Furthermore, the additional use of Hawley retainers may have led to decreased tooth movements compared to the sole use of a 2-point retainer which has to be mentioned as a limitation of this study. Another limitation represents the study design with two independent cohorts for the 1‑ and 3‑year period. For this reason, comparative statements between T1 and T2 cannot be made.

We assume that the combination of the passive stabilization character at the lateral and central incisors by a secured overjet as well as the reliable preservation of the intercanine distance was sufficient for high stability values of the mandibular anterior teeth [6, 12].

Compared to previous investigations of our group, which analyzed posttreatment changes of twistflex retainers, the overall observed position changes of the teeth were reduced in the present cohort treated with 2‑point retainers [32]. A possible explanation for this might be the material properties of the various wires used in the different retention systems. Reported side effects such as the X‑effect and the twist effect seem to occur more frequently with twistflex retainers. These twistflex retainers are less rigid, feature low torsional load transfer, and are discussed to store energy in the area between the composite bonding sites which may be transmitted to the teeth and result in unwanted tooth movement over retention time [9]. However, despite of the newest technologies and developments, a fixed retention concept without any side effects is still not available today [28].

Another important aspect of the current retention concept is a low loss rate, which supports a possible lifelong retention. According to our data, there was no breakage of any of the included retainers. This can be explained by its diameter, which is much larger in comparison with the twistflex retainer [9]. Higher failure rates of multistranded wire retainers compared to round stainless-steel retainers bonded to the canines were also reported by other authors [24–26, 31]. These increased failure rates might be attributed to higher rates of retainer fractures and twisting, as well as to activation of the retainers caused by either deficient bonding or trauma [6, 28].

The present study demonstrated that also in the modern age of orthodontics with innovative digital technologies, the traditional 2‑point steel retainer system presents an effective retention method. In the first few years after treatment, only very minor occlusal adaption has to be expected.
